# Feasibility of the use of *Lentinula edodes* mycelium in terbinafine remediation

**DOI:** 10.1007/s13205-020-02177-6

**Published:** 2020-03-29

**Authors:** Agata Kryczyk-Poprawa, Joanna Piotrowska, Paweł Żmudzki, Włodzimierz Opoka, Bożena Muszyńska

**Affiliations:** 1 Department of Inorganic and Analytical Chemistry, Faculty of Pharmacy, Jagiellonian University Medical College, 9 Medyczna Street, 30-688 Kraków, Poland; 2 Department of Medicinal Chemistry, Faculty of Pharmacy, Jagiellonian University Medical College, 9 Medyczna Street, 30-688 Kraków, Poland; 3 Department of Pharmaceutical Botany, Faculty of Pharmacy, Medical College, Jagiellonian University, Medyczna 9 Street, 30-688 Kraków, Poland

**Keywords:** Terbinafine, Biodegradation, *Lentinula edodes*, Edible mushrooms

## Abstract

A detailed understanding of the fate of xenobiotics introduced into the environment and the mechanisms involved in their biotransformation, biodegradation, and biosorption is essential to improve the efficiency of remediation techniques. Mycoremediation is a form of bioremediation technique that has become increasingly popular in recent years as fungi are known to produce various effective extracellular enzymes that have the potential to neutralize a wide variety of xenobiotics released into the environment. Hence, mycoremediation appears to be a promising technique for the removal of a wide array of toxins and pharmaceutical residues from a damaged environment and wastewater. This study primarily aimed to investigate whether white-rot fungus (*Lentinula edodes*) can be utilized for the bioremediation of common antifungal agent terbinafine, which is mainly available in the market as powder or cream. The cultures of *L. edodes* were cultivated in the medium containing terbinafine powder or terbinafine 1% cream, each at a final concentration of 0.1 mg mL^−1^. The addition of terbinafine in powder form have a negative effect on biomass growth (*p* < 0.05). The total amount of terbinafine in the dry weight of mycelium after culture was estimated to be 7.63 ± 0.45 mg and 12.52 ± 2.46 mg for powder and cream samples, respectively. In addition, there were no traces of terbinafine in any of the samples of medium used for culturing *L. edodes* after the experimental duration period. The biodegradation products of terbinafine were identified for the first time using UPLC/MS/MS. The biodegradation of terbinafine resulted in the loss of 1-naphthylmethanol, which occurred via oxidative deamination, *N*-demethylation, or tert-butyl group hydroxylation. The results of the study demonstrate that *L. edodes* mycelium can be effectively used for the remediation of terbinafine.

## Introduction

Environmental pollution with drugs, pesticides, and personal care products is one of the most challenging issues that the world is facing today. Pharmaceutical compounds play an indispensable role in the treatment and prevention of diseases. Active pharmaceutical ingredients (APIs) can enter the environment as a result of various manufacturing processes involved in the production of medicinal products, following medical diagnosis and therapeutic use of pharmaceuticals for the prevention of diseases, or due to disposal of unused medications. According to Executive Agency for Health and Consumers, the annual worldwide consumption of pharmaceutical compounds is estimated to be about 100,000 tonnes (Mudgal et al. 2013). APIs and their metabolites are identified in sewage, surface water, soils, sludge, and air. Their widespread occurrence and potential adverse effects on ecosystems and human health, mainly associated with long-term exposure to low concentrations of pharmaceuticals, are a subject of public concern (Sousa et al. 2012; Patel et al. 2019). After being released into the environment, active metabolites might further undergo degradation to produce toxic end products, which involves complex processes that significantly depend on various factors (Boxall, 2004). The investigation concerning the potential risk of drugs present in the environment to the aquatic ecosystems is particularly important (Hilton et al. 2003). Therefore, a better understanding of the fate of medicines in the environment is crucial in attempting to achieve effective drug removal from the surroundings. In response to the growing demand for environmental remediation techniques, numerous studies on water and soil remediation processes, including in situ and ex situ methods, have been carried out. Ex situ methods involve isolation, transportation, and disposal of pollutants at a distant and safe place, but this procedure has been proven to be very expensive. In general, technologies based on physicochemical characteristics of pollutants, such as photocatalytic advanced oxidation processes, membrane-based techniques (membrane filtration and reverse osmosis) and chemical and physical adsorption mechanisms, are considered to be highly efficient and cost-effective methods (Kryczyk et al. 2016; Bartolomeu et al. 2018; Warsinger et al. 2018). The recent studies and development in this field are mainly based on the use of nanotechnology, as this novel approach is known to yield better results than the conventional methods. Another possible approach is based on the use of eco-friendly biological processes such as in situ biodegradation. Biological decomposition (bioremediation) is an effective strategy for the removal or transformation of xenobiotics into less toxic forms. Studies conducted worldwide have reported that antifungal drugs (pharmaceutical fungicides) pose a potential threat to the ecosystem. These drugs, particularly those registered for both human and veterinary use, are commonly used as oral or topical agents. This class of drugs could be found in cosmetic products and can be obtained as over-the-counter drugs. In addition, antifungal agents are also widely used in agriculture. Reports of the latest studies establish antifungal drugs as the emerging environmental contaminants (Chen and Ying 2015). The persistence of antifungal drugs in soil and water is highly variable, and it has been observed that some azole antifungals have increased stability and could remain active in some ecological conditions for months (Hof 2001). In Sweden, these agents have been detected in sludge, and the concentrations of clotrimazole, ketoconazole, and econazole are reported to be in the range of 200–1000 µg kg^−1^ dry weight (Lindberg et al. 2010). The occurrence of antifungal agents in the environment has been confirmed in studies conducted locally in many countries (Lindberg et al. 2010; Chen and Ying, 2015; Balakrishna et al. 2017). Terbinafine, an antifungal agent, was detected in the water samples collected from the wells of India at a concentration of more than 1 μg L^−1^ (Fick et al. 2009). Furthermore, this compound was also isolated there from lakes and rivers (Fick et al. 2009). In our previous work, we focused on the mycoremediation of antifungal drugs from the azole group: clotrimazole and bifonazole (Kryczyk-Poprawa et al. 2019). This study primarily aimed to investigate whether white-rot fungus can be effectively utilized for the bioremediation of common antifungal agent terbinafine (in powder and cream forms). Terbinafine hydrochloride, a synthetic allylamine derivative, is widely used for the treatment for fungal infections owing to the fact that it exhibits broad-spectrum antifungal activity and its price is relatively low when compared to other antifungals. Hence, this drug was selected for investigation purpose in the present study. Terbinafine acts by reversible inhibition of squalene epoxidase, an enzyme responsible for ergosterol biosynthesis. This biochemical mechanism is responsible for the fungistatic and fungicidal activity of the drug on fungal mycelia. According to the current medical guidelines, terbinafine is recommended as the first-line treatment for dermatophyte onychomycosis**,** mainly due to its high efficacy and tolerability properties (Ameen et al. 2014). In 2001, about 1.5 million prescriptions for drugs containing terbinafine were issued in the US alone (Davis et al. 2019). Mushroom cultures could also be utilized to remove xenobiotics from the environment (Kryczyk et al. 2017; Dąbrowska et al. 2018). *Lentinula edodes* was selected for the current study because of its unique nutritional and healing properties. The Japanese common name of *L. edodes* is “shitake.” The fruiting bodies of *L. edodes* contain a number of compounds that contribute to its pharmacological effects, mainly immunomodulatory and anticancer properties. The fruiting bodies of this species are rich in bioactive polysaccharides such as β-d-glucans (lentinan), monosaccharides, vitamins, and eritadenine (2(R),3(*R*)-dihydroxy-4-(9-adenyl) butyric acid). These mushrooms also produce many enzymes, such as peroxidase, cellulase, pectinase, xylanase, ligninase, and oxidase. Since *L. edodes* produces oxidative and hydrolytic extracellular enzymes, it can participate in xenobiotic degradation process (Leatham 1985). Additionally, this species was effectively used for the remediation of azole antifungal drugs (Kryczyk-Poprawa et al. 2019). For the above-mentioned reasons, the mycoremediation potential of *L. edodes* mycelia against terbinafine was investigated in in vitro cultures. The medium used for culturing *L. edodes* was enriched with terbinafine powder or terbinafine 1% cream with the final concentration of the investigated drug being 0.1 mg mL^−1^. The biodegradation products of terbinafine were analyzed by UPLC/MS/MS method.

## Materials and methods

### Reagents

Terbinafine hydrochloride (pharmaceutical secondary standard) was obtained from Sigma-Aldrich Corp. (St. Louis, MO, USA). HPLC-grade methanol, acetonitrile, formic acid (98%), chloramphenicol, and dichloromethane were obtained from Merck (Darmstadt, Germany). Chemicals like glucose, maltose extract, casein hydrolysate, l-asparagine, adenine, and yeast extract were purchased from Sigma-Aldrich (St. Louis, MO, USA). NH_4_Cl, KH_2_PO_4_, MgSO_4_⋅7H_2_O, CaCl_2_⋅6H_2_O, FeCl_3_, MnSO_4_⋅H_2_O, and ZnSO_4_⋅7H_2_O were purchased from PPH Golpharm (Kraków, Poland). Water (quadruple-distilled) with a conductivity of less than 1 μS cm^−1^ was obtained using an S2-97A2 distillation apparatus (ChemLand, Stargard Szczeciński, Poland). Terbinafine cream (Terbinafina Ziaja, Ziaja, Poland) was obtained from a local pharmacy store.

### Materials for analysis

**Fig. 1:**
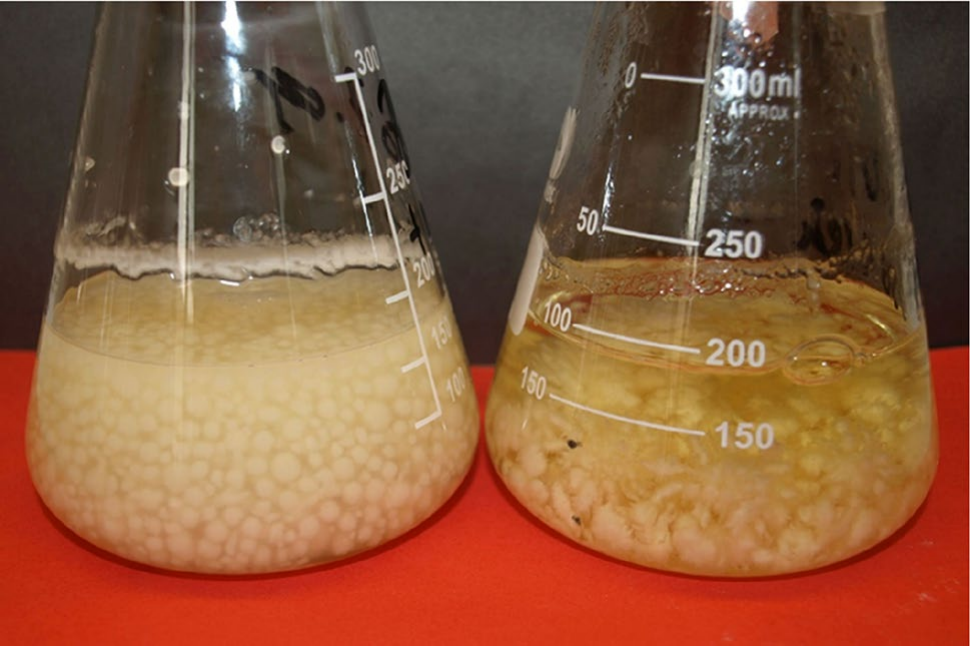
*L. edodes *in vitro cultures enriched with the addition of 2.5 g of terbinafine 1% cream or 25 mg terbinafine powder per 250 mL of the Oddoux medium (picture taken by Agata Kryczyk-Poprawa)

For the study, commercially available fruiting bodies of *L. edodes* (Berk.) (Pegler (mushroom) were bought at a supermarket in Poland (2016). The taxonomic identification of the fungus was performed using MycoKey 4.1 software (https://www.mycokey.com) by an expert named Muszyńska. The representative samples of the material to be used for further studies were stored at the Department of Pharmaceutical Botany, Jagiellonian University Medical College, Kraków, Poland. Fragments of the hymenial part of fruiting bodies were selected to prepare the mycelial culture of *L. edodes*. First, the fragments of the fruiting bodies were mixed with sterile redistilled water and transferred to the BD Sabouraud agar supplemented with chloramphenicol (under laminar airflow). Then, the cultures were incubated in a thermostat (ST500/B/40 Pol-Eko-Apparatus) at 23 °C ± 2 °C for 2 weeks. Microscopic analysis of the obtained mycelium of *L. edodes* was performed by Muszyńska. It showed the homogeneous nature of hyphae, which was free from contamination with other strains of fungi or bacteria. The mycelia obtained from the solid medium were used to prepare initial cultures that were grown on liquid Oddoux medium (Oddoux 1957). The cultures were shaken on a rotary shaker (ALTEL, Poland) at a rate of 140 rpm and incubated at a temperature of 23 °C ± 2 °C under 12-h light (900 lx)/12-h dark cycle. The cultures of *L. edodes* were maintained for two weeks and later subcultured (Fig. 1).

The obtained *L. edodes* mycelium was analyzed by PCR method. GenBank Accession Number SUB6791903 Seq 1 MN907099 was assigned to the nucleotide sequence determined in this study.

### Experimental mycelial cultures of *L. edodes*

For mycoremediation studies, 25 mg of terbinafine powder or 2.5 g of terbinafine 1% cream was added into 250 mL of sterile liquid medium inoculated with a mycelial culture of *L. edodes.* Terbinafine 1% cream (Ziaja) containing isopropyl myristate, cetyl alcohol, stearyl alcohol, sorbitan stearate (Type 50), cetyl palmitate (15), polysorbate 60, benzyl alcohol, sodium hydroxide, and purified water was used. Moreover, control samples were prepared without the addition of terbinafine. After inoculation, the cultures were incubated for 14 days. Then, the biomass of *L. edodes* was separated from the medium, rinsed with redistilled water, and then freeze-dried using a lyophilizer (FreeZone 4.5, Labconco).

### RP-HPLC analysis

Five grams of the lyophilized mushroom culture (mycelium obtained from in vitro cultures) was ground in a mortar and then extracted with a mixture of methanol and dichloromethane (75:25, v/v) in an ultrasonic bath at 49 kHz for 30 min (Sonic-2, Polsonic). The obtained extracts (300 mL) were evaporated to dryness using a rotary vacuum evaporator at 22 °C ± 2 °C, and then the dried extracts were dissolved in methanol and filtered through a 0.2-μm filter. The concentration of terbinafine was determined using RP-HPLC according to the procedure developed by Tagliari (2010). Briefly, the separation was carried out on Hitachi HPLC apparatus (Merck, Tokyo, Japan) equipped with an L-7100 pump, Purospher® RP-C_18_ (200 mm × 4 mm , 5 μm) column (Merck, Tokyo, Japan), using methanol–water (95:5, v/v) as a mobile phase, and a flow rate of 1 mL min^−1^ with UV detection at *λ* = 254 nm. The qualitative analysis of terbinafine was done by comparing the retention times of the peaks in the samples with the retention times of standard. To confirm the presence of terbinafine in the tested extracts, a standard solution was added to the samples. The presence of the tested compound in the sample was indicated by an increase in the peak height for the appropriate retention time. In addition, MS/MS analysis was also performed. The quantitative analysis of terbinafine was carried out using the calibration curve method where the concentration of the standard drug was in the range from 0.01 to 0.1 mg mL^−1^.

### UPLC/MS/MS analysis

About 200 μL of the sample was evaporated and diluted in 300 μL of water with the addition of 1% formic acid. The samples were applied to the SPE cartridges (Waters Oasis HLB Cartridge, USA) and washed with 1 mL of water, followed by the addition of 1 mL of methanol–water (50:50, v/v) mixture, and then eluted with 1 mL of methanol. The prepared samples were injected into the UPLC/MS/MS system for analysis. The UPLC/MS/MS system consisted of a Waters ACQUITY® UPLC® (Waters Corporation, Milford, MA, USA) coupled to a Waters TQD mass spectrometer (electrospray ionization mode ESI-tandem quadrupole). Chromatographic separation was carried out using Acquity UPLC BEH (bridged ethyl hybrid) C18 column, 2.1 × 100 mm and 1.7 µm particle size, equipped with Acquity UPLC BEH C18 VanGuard pre-column, 2.1 × 5 mm and 1.7 µm particle size. The column was maintained at 40 °C, and eluted under gradient conditions using 95–0% of eluent A for 10 min, at a flow rate of 0.3 mL min^−1^. Eluent A was composed of water/formic acid (0.1%, v/v), and eluent B was composed of acetonitrile/formic acid (0.1%, v/v). Chromatograms were recorded using Waters eλ PDA detector. Spectra were evaluated in 200–700 nm range with 1.2-nm resolution at a sampling rate of 20 points/s. Mass spectrometry detection settings of Waters TQD mass spectrometer were as follows: source temperature 150 °C, desolvation temperature 350 °C, desolvation gas flow rate 600 L h^−1^, cone gas flow rate 100 L h^−1^, capillary potential 3.00 kV, and cone potential 6 V. Nitrogen gas was used as both nebulizing and drying agent. The data were recorded in the scan mode ranging from 50 to 1000 *m*/*z* in time intervals of 0.5 s; eight scans were summed up to obtain the final spectrum. The data acquisition software used was MassLynx V 4.1 (Waters).

### Statistical analysis

Statistical analyses were conducted using Microsoft Excel 2010 and GraphPad Prism v3.02 (GraphPad Software, San Diego, CA, USA). Three replicates were used for each sample. Results are expressed as means and standard deviation (SD). One-way analysis of variance test with Tukey’s post hoc test of multiple comparisons or Mann–Whitney's nonparametric test was applied. Values were considered significantly different at *p* < 0.05. In silico toxicity risk profile of biotransformation products was predicted using OSIRIS Property Explorer (access: https://www.organic-chemistry.org/prog/peo/).

## Results and discussion

Biodegradation is a biochemical process that leads to the degradation and recycling of xenobiotics. This process primarily involves bacteria, fungi, and algae, and their activity leads to the decomposition of the initial substance into simpler compounds, which do not usually exhibit toxic effects. Due to increasing risks associated with environmental pollution, novel methods showing increased bioremediation efficiency and new possibilities of its application are being explored. It should be emphasized that mycoremediation has become an increasingly popular method for the removal of pollutants from the environment, and, therefore, studies to identify new fungal species with the potential to degrade xenobiotic compounds to evaluate the effectiveness of mushroom-based remediation techniques are highly warranted. The knowledge regarding the biodegradation and biotransformation of antifungal drugs by the mycelia obtained from in vitro cultures is one of the essential elements that prompted us to select white-rot fungus, *L. edodes,* for mycoremediation of the xenobiotic in our study.

**Fig. 2:**
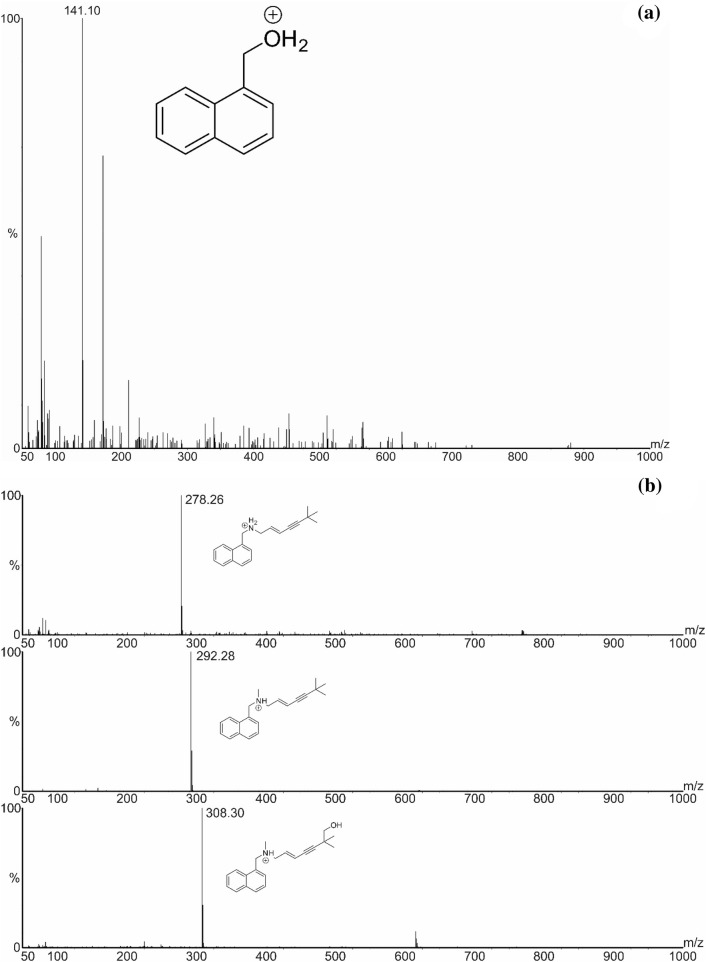
MS/MS spectra of biodegradation products of terbinafine: **a** TP-1 and **b** TP-12, terbinafine, and TP-7

Terbinafine is metabolized in animals and humans (Schatz et al. 1986). It is absorbed from the gastrointestinal tract in more than 70% of the individuals after oral administration, following intake of 250 mg of the drug, and its maximum plasma concentration is noticed within 1.5 h (Elewski et al. 2005). The drug accumulates in adipose tissue and keratin-rich tissues (dermis, hair, and nails), wherein effective fungicidal concentration is observed. It is metabolized in the presence of CYP isoenzymes to metabolites that lack antifungal activity. Fifteen liver metabolites of terbinafine have been described during preclinical and clinical studies (Iverson and Uetrecht, 2001). The hepatic metabolism of terbinafine mainly takes place through *N*-demethylation, deamination, and oxidation of the alkyl side chain (Vickers et al. 1999). The present study was undertaken to compare the biodegradation products of terbinafine, its hepatic metabolites, and its photoproducts formed under UVA irradiation. The identification of the degradation products of terbinafine in the mycelium obtained from in vitro cultures of *L. edodes* was performed on the basis of UPLC/MS/MS analysis. The proposed structures of the degradation products of terbinafine are shown in Fig. 2. Two biodegradation products of terbinafine (TP-1 and TP-7) have been previously described. TP-1 and TP-7 were formed during degradation of terbinafine in the presence of UVA irradiation (Kryczyk-Poprawa et al. 2019). The biodegradation of terbinafine resulted in the loss of 1-naphthylmethanol, which occurred via oxidative deamination, *N*-demethylation, or tert-butyl group hydroxylation. The structures of potential products of terbinafine degradation identified in the mycelium obtained from in vitro cultures of *L. edodes* showed the following properties: TP-1 produced a [M + H]^+^ at *m/z* 141.1, TP-7 produced a [M + H]^+^ at *m/z* 308, and TP-12—identified for the first time—produced a [M + H]^+^ at *m/z* 278. TP-12 was formed as a result of N-demethylation of terbinafine to desmethyl terbinafine. This compound further forms 6, 6-dimethyl-2-hepten-4-ynal in the liver—a compound that can initiate idiosyncratic drug-induced liver toxicity (Iverson and Uetrecht 2001; Barnette et al. 2019). After previous application of β-glucuronidase hydrolysis in urine and plasma, the metabolite TP-7 was determined which was also identified in mycelium (Zehender et al. 1995).

Many ecotoxicological test systems have been introduced to study the effect of xenobiotics on the aquatic organisms (Küster and Adler 2014). The toxicity studies of terbinafine were carried out in the green algae *Pseudokirchneriella subcapitata*. Terbinafine is listed in the Swedish Environmental Classification of Pharmaceuticals as the only antifungal drug that poses moderate risk to the environment alongside other compounds such as chlorhexidine, mycophenolic acid and norethisterone (Graae and Pålsson 2018). The harmful effects of terbinafine hydrochloride have been described in Pharmacopeia, in Safety Data Sheet Sect. 2 (Hazards Identification), where it is mentioned that terbinafine is very toxic for aquatic organisms (British Pharmacopoeia Commission 2015). There are also data on the genotoxic effects of terbinafine (Chien et al. 2012; Tolomeotti et al. 2015). We next aimed to evaluate the toxicity–risk predictions of terbinafine and its degradation products using OSIRIS Property Explorer. The toxicity risk assessment was done based on their ability to induce mutagenicity, tumorigenicity, irritation, and reproductive effects (Table 1). The in silico experiments revealed that terbinafine and its degradation products theoretically displayed high tumorigenic activity, which can be attributed to the presence of 1-naphthyl fragment. TP-1, a product of oxidative deamination, led to the formation of 1-naphthylmethanol which demonstrated medium mutagenic activity; hence, further studies aiming to evaluate its safety profile are required.

**Table 1:** Toxicity risks of terbinafine and its biodegradation products determined in *L. edodes* mycelium obtained from in vitro cultures in which media were enriched with terbinafine and predicted by OSIRIS Property Explorer

Compound	Mutagenic	Tumorigenic	Irritant	Reproductive effects
Terbinafine	–	High-risk fragment (1-naphthyl)	–	–
TP-1	Medium-risk fragment (1-naphthylmethanol)	High-risk fragment (1-naphthyl)	–	–
TP-7	–	High-risk fragment (1-naphthyl)	–	–
TP-12	–	High-risk fragment (1-naphthyl)	–	–

**Fig. 3:**
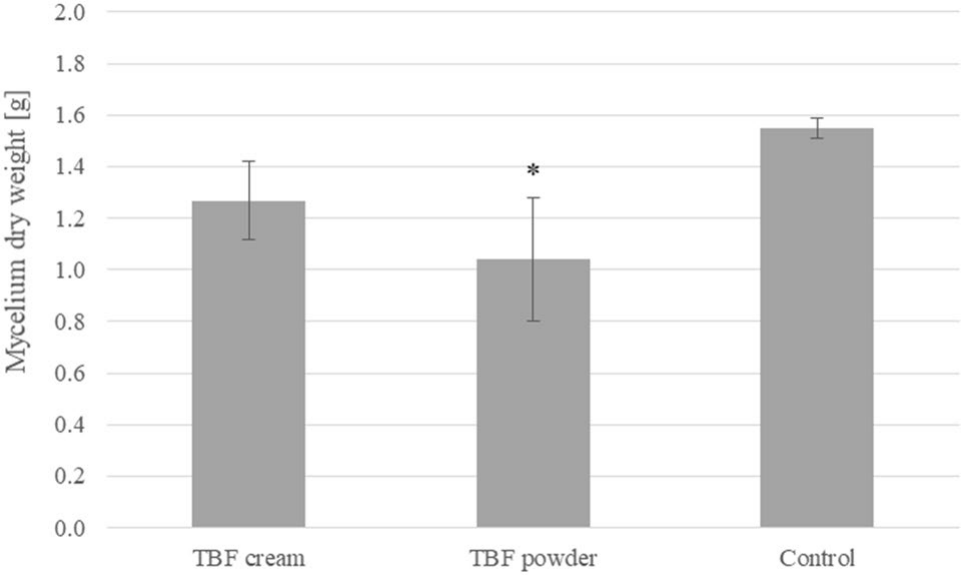
Comparison of dry matter of Lentinula edodes mycelium obtained from in vitro cultures enriched with the addition of 2.5 g of terbinafine 1% cream or 25 mg of terbinafine powder per 250 mL of the medium. Statistical analysis: one-way analysis of variance (ANOVA), followed by Tukey’s test. Significant difference compared to the control group (without terbinafine addition): * p < 0.05, *n* = 3

Figure 3 shows a comparison of dry biomass obtained from in vitro cultures of *L. edodes* enriched with terbinafine (2.5 g of terbinafine 1% cream or 25 mg of terbinafine powder) and control (without the addition of terbinafine). The dry weight of mycelium showed statistically significant differences between the control and the fungal sample supplemented with terbinafine powder (*p* < 0.05). These differences may result from terbinafine concentration in the medium. The addition of terbinafine in the form of powder, which shows poor solubility in the water, probably allows for immediate availability of the drug for the remediation process, which could have a negative effect on biomass growth. In the case of cream, the form of the drug itself slows down the rate of release of terbinafine into the medium and, thus, limits its availability for the remediation process.

**Fig. 4:**
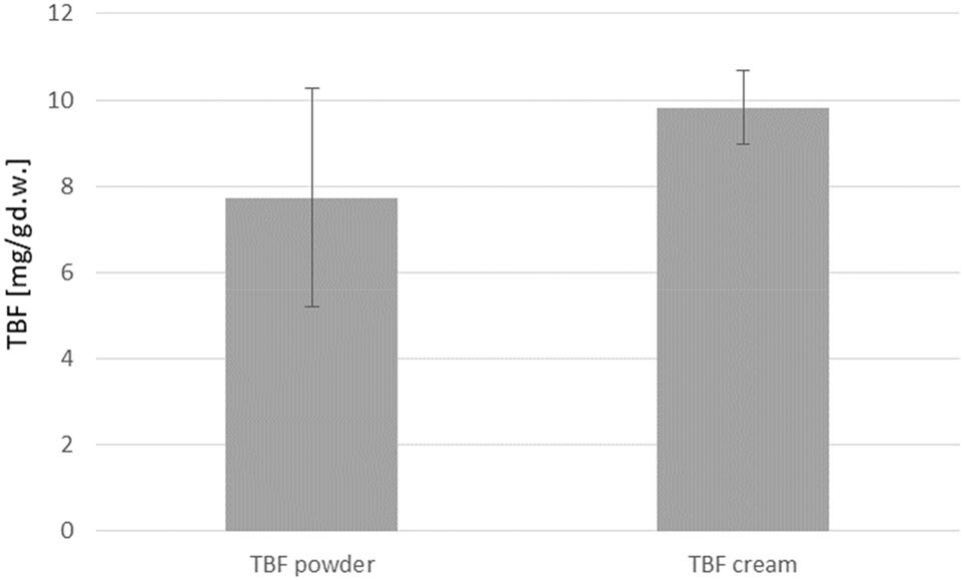
Terbinafine content in 1 g of dry matter obtained from in vitro cultures *of Lentinula edodes* enriched with the addition of 25 mg of terbinafine powder or 2.5 g of terbinafine 1% cream per 250 mL of the medium. Statistical analysis: Mann–Whitney' nonparametric test: NS, *n* = 3

The next stage of the study was to compare of the amount of the tested compound present per 1 g of dry matter of the mycelium in different samples after the experimental duration period. The calculation took into account the mass of dry mycelium obtained after 4 weeks of cultivation. Figure 4 presents terbinafine concentrations in mycelium obtained from in vitro cultures of *L. edodes* on Oddoux medium enriched with 25 mg of terbinafine or 2.5 g of terbinafine 1% cream per 250 mL of the medium, respectively. There were no statistically significant (*p* < 0.05) differences in the concentration of terbinafine in the mycelium cultured in the medium containing different forms of the drug. The content of terbinafine in the medium was below the limit of quantification in all the analyzed samples, which indicates that the drug was successfully degraded by the fungus. Therefore, mycoremediation can serve as an alternative approach to traditional remediation techniques.

The total content of the investigated drug in *L. edodes* mycelium was calculated on the basis of the terbinafine content determined in 1 g of dry matter obtained from *L. edodes *in vitro cultures and the dry weight of the examined mycelium. The total terbinafine content in *L. edodes* mycelium, obtained from in vitro cultures, in which media were enriched with terbinafine in powder or cream form at a concentration of 0.1 mg mL^−1^ was 7.63 ± 0.45 mg and 12.52 ± 2.46 mg, respectively. The use of two different forms of the drug allowed to demonstrate the impact of drug form on the process of remediation by *L. edodes*. A higher amount of terbinafine was determined in the dry matter obtained from in vitro cultures of *L. edodes* supplemented with 1% cream. In the case of powder, the amount of terbinafine determined in the mycelium obtained from in vitro cultures of *L. edodes* and its absence in the medium indicate a more advanced process of biodegradation.

## Conclusion

Many reports have been published concerning the role of antifungal agents in causing environmental pollution. The present study focuses on the evaluation of the potential of *L. edodes* to accumulate or/and degrade the common antifungal drug terbinafine. In vitro culturing of *L. edodes* in the presence of the drug, thanks to the application of reproducible culture conditions, and examining the amount of the drug in the mycelium after culture allowed for the assessment of the remediation process of the examined compound. The study results demonstrates that *L. edodes* can be effectively used for the bioremediation of terbinafine.
